# Influence of Some Hydrocolloids and Sterilization Conditions on the Physical Properties of Texture-Modified Foods Developed for the Swallow Training of Dysphagia Patients

**DOI:** 10.3390/foods12193676

**Published:** 2023-10-07

**Authors:** Thitiwat Limampai, Rarisara Impaprasert, Worapot Suntornsuk

**Affiliations:** Department of Microbiology, Faculty of Science, King Mongkut’s University of Technology Thonburi, Bangkok 10140, Thailand; thitiwat.lim@kmutt.ac.th (T.L.); worapot.sun@kmutt.ac.th (W.S.)

**Keywords:** dysphagia, swallow training, agar, κ-carrageenan, gelatin, konjac glucomannan, xanthan gum

## Abstract

This research aimed to develop jelly soup for dysphagia patients at the International Dysphagia Diet Standardization Initiative (IDDSI) Framework levels 4 (puree) and 5 (minced and moist), who require swallow training to regain normal swallowing ability due to neurological issues. The study comprised three main parts: (1) an investigation of hydrocolloid types and concentrations for texture-modified foods to aid dysphagia patients during training; (2) a study of sterilization conditions and ascorbic acid’s impact on physical properties (e.g., texture, viscosity, color) of the texture-modified foods; and (3) an evaluation of changes in physical, chemical, and microbial properties of the product during storage. Results revealed that the ideal recipe involved using pork bone broth with 1% κ-carrageenan for texture modification, which closely matched the properties of hospital jelly samples in terms of hardness, adhesiveness, and viscosity. Sterilization at 110 °C for 109 min effectively eliminated microorganisms without affecting the product’s appearance or texture, albeit causing a slight increase in brownness. Adding ascorbic acid helped to prevent the Maillard reaction but reduced the gel strength of the sample and induced milk protein denaturation, leading to aggregation. During storage at room temperature for 9 weeks, the product became browner and less firm. Notably, no bacteria were detected throughout this period. In conclusion, this heating process is suitable for producing jelly soup to support swallow training for dysphagia patients with neurological problems. It offers invaluable assistance in their daily training to regain normal swallowing function.

## 1. Introduction

Currently, the elderly population is rapidly growing in many parts of the world [1]. A common problem among the elderly is choking while eating. Elderly people often suffer from a neurological disease known as Parkinson’s disease. The largest number of Parkinson’s disease (PD) patients was seen in the groups aged more than 65 years, and the percentage rapidly increased in the population aged more than 80 years. The Global Burden of Disease (GBD) study reported that incident cases of PD were 1.02 million in 2017. There were 6.1 million PD patients reported in 2016 globally [2]. Additionally, 82 percent of these patients experience choking and swallowing problems [3], similar to stroke patients. Food choking is the primary cause of an increased risk of lung infections, and it is also the leading cause of death in Parkinson’s patients [4,5]. Currently, the classification of food for patients with swallowing difficulties is categorized into eight levels, (0) thin, (1) slightly thick, (2) mildly thick, (3) moderately thick (liquidized), (4) extremely thick (pureed), (5) minced and moist, (6) soft and bite-sized, and (7) regular (easy to chew), by the International Dysphagia Diet Standardization Initiative (IDDSI) Framework [6]. A dysphagia mechanically altered diet consists of liquid food that is highly moist, with a soft texture that can be easily swallowed (level 4–5). A dysphagia advanced diet comprises food that is as stiff and sticky as normal food but in smaller pieces (level 6).

Swallowing problems are typically evaluated by measuring the swallowing ability according to a manual, under the supervision of rehabilitation medicine, in accordance with guidelines for promoting safe swallowing [7]. This ability can be improved from the measured level, enabling a return to normal food intake. In Thailand, food innovations for patients with swallowing problems were initiated in 2011. Most of the innovative food has a gel-like appearance but is softer and smoother than a regular diet, allowing patients to chew and swallow without choking or getting the food stuck in the throat. However, this food is still not suitable for patients whose dysphagia stems from Parkinson’s disease. In 2015, researchers invented rice-berry rice jelly for the elderly with dysphagia, but the specificity of the diet and its suitability for patients’ levels of dysphagia were not determined [8].

Foods designed to improve swallowing typically exist in the form of ready-to-eat or general diet foods, following the recommended dietary characteristics of each level as indicated by the International Dysphagia Diet Standardization Initiative (IDDSI) Framework [6]. However, these foods are not suitable for daily consumption, as they are primarily designed as desserts, such as jelly and pudding, etc. Furthermore, there are irregularities in food texture preparation, as per the IDDSI standards.

In recent years, there have been efforts in researching food for dysphagia patients in various studies. For example, the use of carrot puree with 2% gelatin, 2% guar gum, or 2% xanthan gum [9]; the use of 88.59% beef, 0.89% carboxy methyl cellulose, and 0.02% tapioca starch in beef patties as an IDDSI level 6 food [10]; the use of black fungus with 0.9% xanthan gum as an IDDSI level 5 diet [11]; the production of sea buckthorn jelly using 3.01% guar gum, 5.74% xanthan gum, and 11.38% locust bean gum, resulting in a smooth texture that can be easily chewed by the elderly [12]; the use of calcium salts-induced mung bean starch-flaxseed protein composite gels as level 5 and level 6 dysphagia foods [13]; and the use of rice starch, perilla seed oil, and whey isolate protein in level 4 foods in the IDDSI framework [14]. However, it was found that the aforementioned research primarily focused on shaping food using molds or employing 3D food printers to investigate the texture characteristics of food. There is still a lack of research related to microbial sterilization systems and the study of food changes during storage. Therefore, this research project was undertaken. The objective of this research was to produce ready-to-eat food for the swallow training of dysphagia stemming from neurological diseases. Various hydrocolloid substances were used to enhance the texture of food for practicing swallowing ability in daily training. The texture-modified food, with a similar viscosity at the apparent shear rate and similar texture profile analysis values to a reference used in hospitals, was examined to select a heat sterilization method that would not affect its physical characteristics. Monitoring of the texture changes during storage was also undertaken.

## 2. Materials and Methods

### 2.1. Chemicals

The hydrocolloids used in this study were agar, κ-carrageenan, gelatin, konjac glucomannan, and xanthan gum from Union Chemical (Bangkok, Thailand), and plate count agar from Himedia Laboratories Pvt. Ltd. (Ambernath, Maharashtra, India).

### 2.2. Sample Preparation and Food Reference

Ingredients for the preparation of texture-modified foods were obtained from a local market. These included 1 kg of pork bone, 10.32 g of coriander root, 43.72 g of garlic, 2.18 g of pepper, 13.60 g of refined sugar, and 2.88 g of salt. They were then mixed and stewed with 3 L of water until the volume decreased to 1 L. Afterward, solid residues were removed through filtering, and the resulting broth was mixed with 176 mL of unsweetened condensed milk (Carnation^®^, F&N Dairies Co., Ltd., Bangkok, Thailand). The hydrocolloids were subsequently added to the broth, homogenized for 15 min by using a homogenizer, and pasteurized at 72 °C for 30 min. Different types and concentrations of hydrocolloids were used in this experiment, as shown in Table 1. Samples (200 mL) were placed in PET plastic cups and refrigerated at 4 ± 1 °C for 24 h before conducting measurements for texture profile analysis and viscosity.

A Richese^®^ jelly (Sahapat Co., Ltd., Bangkok, Thailand) was used as a reference sample in this experiment. This jelly is commonly used in hospitals to train patients with level 4 and 5 swallowing problems according to IDDSI standards.

### 2.3. Sample Measurement

#### 2.3.1. Viscosity

The viscosity of the mashed sample (5 ± 0.5 g) was measured using an Advanced Rheometer MCR 150 (Anton Paar GmbH, Graz, Austria) with a CP 50-2/Q1 measuring cone set at a gap of 0.049 mm. A continuous shear rate ramp ranging from 1 to 150 s^−1^ at 25 °C was applied, and the temperature was maintained using an external water bath. Apparent viscosity values at a shear rate of 50 s^−1^ were selected for comparison among samples, with Richese^®^ jelly (Sahapat Co., Ltd., Bangkok, Thailand) serving as the reference [15].

#### 2.3.2. Mechanical Properties

The samples were melted, packed into a cup with a diameter of 4 cm and a height of 2 cm, and then refrigerated at 4 ± 1 °C for 24 h before testing. Texture profile analysis was conducted using a TA.XTplus Texture Analyzer (Stable Micro Systems Ltd., Godalming, Surrey, UK). A two-bite compression test was performed using a P6 probe at a speed of 2 mm/s over a distance of 15 mm. The Micro Systems software was employed to extract data from the texture profile analysis curve for primary and secondary parameters, including hardness, adhesiveness, cohesiveness, and springiness.

### 2.4. Sterilization Study

The samples, possessing viscosity and mechanical properties akin to the reference jelly, both with and without the addition of ascorbic acid (0.1% *w*/*v*), were placed in laminated retort pouches and sealed tightly. The horizontal stationary retorting system (Km Grand Pack Co., Ltd., Samut Prakan, Thailand) was used for sterilization. All of the packages were heated in a pressurized retorting system at the specified temperature–time profiles (110 °C—109 min, 121 °C—37 min, and 125 °C—11 min). These sterilization conditions were established following the protocol by Fernandez et al. [16]. Subsequently, both the fresh and sterilized samples were stored in a refrigerated environment at 4 ± 1 °C for 24 h prior to conducting analyses for texture profile, viscosity, color, and visual appearance. Additionally, a microbiological assessment of the samples was carried out using the total plate count method.

### 2.5. Microbiological Study

The sterilized samples were subjected to a bacterial count assessment using the total plate agar method, following the guidelines outlined in the BAM protocol [17].

### 2.6. Color Measurement

Sample colors were conducted using a colorimeter (UltraScan Pro, Hunter Lab, Victoria, Australia), with visual observation also employed for monitoring. Color values of sample were measured and expressed in terms of CIE, L* (lightness), a* (redness), and b* (yellowness), with the instrument standardized using standard color tiles, namely white (X: 82.18, Y: 87.18, Z: 94.03) and green (X: 18.69, Y: 24.69, Z: 21.05), under the D65/10° illuminant mode and SPIN LAV observer. Subsequently, the sterilized mashed sample (20 ± 1 g) was packed into a Quartz cell (Transmission cell), filling at least ¾ of the cell volume, and then the color value was measured in the CIE Lab* system through three repeated measurements. A Browning assessment was carried out by calculating the formula as follows [18]:

Browning Index (BI) = [100 (x − 0.31)]/0.172(1)

where x = (a* + 1.75L*)/(5.645L* + a* − 3.012b*).

### 2.7. Stability Study

The sterilized selected sample was stored at room temperature (30 ± 2 °C) for a duration of 9 weeks to facilitate the determination of the texture profile analysis, viscosity, color, pH, and microbiological study. All parameters were measured weekly following the AOAC method [19].

### 2.8. Data Analysis

All the collected data were analyzed using SPSS software [20]. A one-way ANOVA was performed on the rheology and texture profile analysis data to ascertain significant differences among treatments (*p* ≤ 0.05). Subsequently, Duncan’s multiple range test was employed to discern variations among the samples.

## 3. Results

### 3.1. Effects of Hydrocolloids on the Texture-Modified Foods

#### 3.1.1. Physical Properties

The hardness, adhesiveness, springiness, and cohesiveness values of the texture-modified foods treated with different types and concentrations of hydrocolloids are presented in Table 2. κ-Carrageenan significantly influenced the physical properties of the texture-modified foods. However, xanthan gum, at all studied concentrations, as well as low concentrations of glucomannan and gelatin, were unable to form gels in the texture-modified foods. Among the hydrocolloids at the same concentrations, the texture-modified foods treated with κ-carrageenan exhibited the highest hardness value.

κ-Carrageenan, a polysaccharide extracted from red seaweed, is a commonly used food additive. When added to milk, κ-carrageenan can interact with the milk’s casein proteins, resulting in the formation of a stronger, gel-like structure. This phenomenon, known as synergistic interaction, arises due to κ-carrageenan’s high affinity for casein. Casein, a milk protein, is responsible for milk’s opaque appearance. When milk is heated or acidified, casein molecules can create a loose network, leading to milk coagulation and the development of a soft, gel-like structure. κ-Carrageenan reinforces the casein network by creating additional bonds between casein molecules. Aligned with the research of Balakrishnan et al. [21] and Tang et al. [22], where casein remained stable up to 100 °C at a neutral pH, casein’s DSC thermograms showed an endothermic peak at 115.8 °C. Adding κ-carrageenan raised this peak to 127.2 °C. This suggests that κ-carrageenan increases casein’s thermal denaturation temperature, enhancing its thermal stability. κ-Carrageenan’s presence fosters interactions, including hydrogen bonding and electrostatic interaction, between the two, leading to protein-rich domain formation. κ-Carrageenan carries a negative charge, whereas casein holds a positive charge. This charge disparity enables κ-carrageenan molecules to bind to casein molecules, resulting in a more stable structure.

Apart from its interaction with casein, κ-carrageenan possesses its own gelling properties, rendering it a valuable ingredient in various food products. The synergy between κ-carrageenan and casein can produce a more robust and stable structure in items like dairy products, puddings, and processed meats [23,24].

Furthermore, proteins in foods cannot facilitate the gel formation of texture-modified foods treated with agar, given that agar contains a comparatively lower quantity of sulfur groups in its structure. As a result, its reactivity with food proteins is reduced or negligible [25]. On the other hand, the gel formation of gelatin is primarily influenced by its concentration and forming temperature. The proteins in the food matrix do not significantly affect the hardness and stability of texture-modified foods treated with gelatin [26].

As indicated by the data presented in Table 2, texture-modified foods treated with 5% agar, 1% κ-carrageenan, and 5% gelatin exhibited similar hardness values to the reference sample (*p* > 0.05). However, the adhesiveness value of the texture-modified foods treated with 1% κ-carrageenan did not display any statistically significant difference (*p* > 0.05) when compared to the reference sample.

The physical characteristics of gel formation and syneresis of the sample are described in Table 3. From the observations obtained during the experiment, it was found that only the use of 5% konjac glucomannan or 1% carrageenan provided a texture closely resembling the reference sample and exhibited the desired gel-like characteristics, specifically a soft gel without syneresis. However, the use of 5% konjac glucomannan resulted in a texture that was excessively adhesive in the mouth. Therefore, the most suitable condition was achieved by using 1% carrageenan.

In summary, the sample obtained from the experiment possessed a moderately soft and chewable texture. Increasing the concentration of the hydrocolloid notably enhanced the gel strength (hardness) of the sample, albeit at the cost of reduced springiness. Nevertheless, this heightened concentration did not appear to substantially impact its cohesiveness properties. The experiment’s findings demonstrate that xanthan gum is incapable of forming a gel on its own at any concentration, whereas konjac glucomannan can produce a gel when utilized at concentrations exceeding 5%.

#### 3.1.2. Viscosity of the Sample before the Sterilization Process

Hydrocolloids serve two primary purposes in the food industry: enhancing consistency and facilitating gel formation. They are employed as thickening and gelling agents, with their effectiveness influenced by the specific hydrocolloid type, concentration, pH, temperature, and the nature of the food products. This application leads to extended shelf life and improved food quality. Various gums like xanthan, konjac glucomannan, guar, and starch are utilized as thickeners in soups, sauces, additives, salad dressings, and other food items. Gelling agents such as pectin, alginate, agar, gelatin, and carrageenan find predominant use in products like jellies, jams, and ice cream [27]. Several key factors significantly impact the viscosity of polymer solutions. The first factor is the molecular mass of the polymer. Viscosity becomes more dependent on shear rate as the molecular mass increases, and simultaneously the shear rate at which shear thinning occurs shifts to lower values with higher molecular mass. The second critical factor is the hydrodynamic size. Linear and rigid molecules yield solutions with considerably higher viscosity compared to highly branched and flexible polymers of the same molecular mass due to their larger hydrodynamic size. The third essential factor is the charge of the polymers. The presence of charged functional groups along the polymer backbone results in solutions with higher viscosity than those composed of non-ionic polymers with similar molecular masses. This is primarily because charged polymers adopt a more expanded configuration in the solution due to intramolecular charge repulsions. Adjusting either the pH or the ionic strength of the solution can lead to a significant reduction in viscosity by promoting the aggregation of polymer chains through a decrease in the degree of dissociation of the charged groups [28].

**Figure 1:**
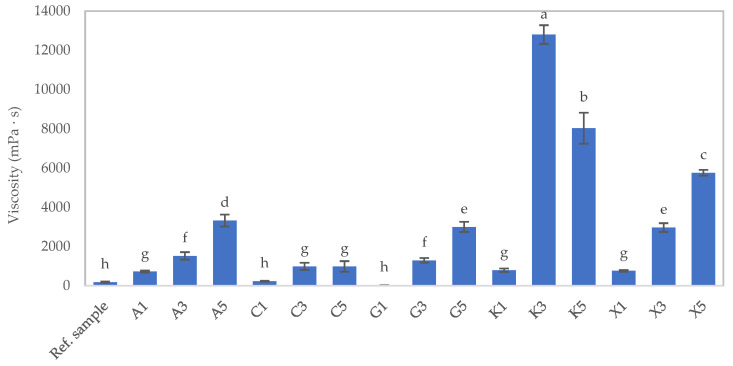
The viscosity of texture-modified foods, subjected to various types and concentrations of hydrocolloids at a shear rate of 50 s^−1^ (refer to sample abbreviations in Table 1), is illustrated in the graph. Different lowercase letters on the bars signify statistically significant differences between the samples (*p* ≤ 0.05).

From the experimental results in Figure 1, the samples can be divided into two groups: the first group consists of hydrocolloids with low viscosity and the ability to form gels, and includes agar, gelatin, and carrageenan, and the second group is characterized by high viscosity and no ability to form gels, and includes konjac glucomannan and xanthan gum. All hydrocolloids, across various concentrations, influenced the viscosity of the texture-modified foods. Elevated concentrations of all hydrocolloids led to increased viscosity in the texture-modified foods. The viscosity of the texture-modified foods treated with 1% κ-carrageenan and 1% gelatin did not exhibit a statistically significant difference (*p* > 0.05) compared to the value obtained from the reference sample. Nevertheless, it is important to highlight that gels made from gelatin can liquefy at body temperature, potentially presenting a choking hazard for patients during swallowing. Consequently, its suitability for daily consumption is questionable. On the other hand, 1% κ-carrageenan emerges as a potentially viable option for everyday use, as it possesses hardness, adhesiveness, and viscosity values that closely align with those of the reference sample.

Based on the results of the texture and viscosity property experiments of the samples in Table 2 and Figure 1, it can be concluded that only the sample containing 1% κ-carrageenan exhibited a texture profile (hardness and adhesiveness) and viscosity properties closest to the reference sample. For the other samples, apart from the 1% κ-carrageenan sample, significant differences in both texture profile and viscosity properties were observed. The agar-based sample had the ability to adhere to the outer surface and had excessive hardness, resulting in potential choking or swallowing difficulties. As for the samples with κ-carrageenan concentrations greater than 1%, there was a tendency to cause swallowing difficulties, similar to the agar sample. Although the sample containing 1% gelatin showed test results similar to the reference sample, it still experienced dissolution issues at body temperature, which could lead to swallowing difficulties. As for the samples containing glucomannan and xanthan gum, they had significantly higher viscosity values compared to the reference sample, making them difficult for patients to swallow.

### 3.2. Effects of Sterilization Conditions on the Texture-Modified Foods

#### 3.2.1. Physical Properties

**Figure 2:**
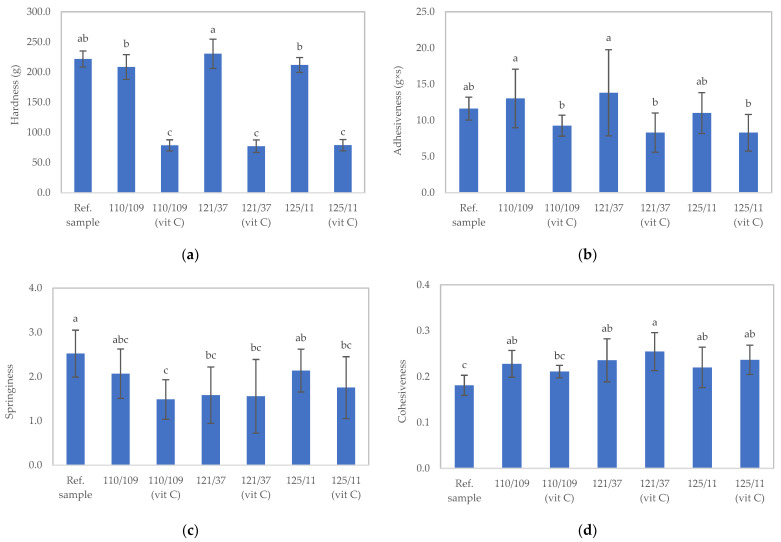
Texture profile analysis of texture-modified foods following sterilization under distinct conditions: (**a**) hardness, (**b**) adhesiveness, (**c**) springiness, and (**d**) cohesiveness. Sterilization conditions: 110/109: 110 °C for 109 min without ascorbic acid; 121/37: 121 °C for 37 min without ascorbic acid; 125/11: 125 °C for 11 min without ascorbic acid; 110/109 (Vit C): 110 °C for 109 min with 0.1% (*w*/*v*) ascorbic acid addition; 121/37 (Vit C): 121 °C for 37 min with 0.1% (*w*/*v*) ascorbic acid addition; 125/11 (Vit C): 125 °C for 11 min with 0.1% (*w*/*v*) ascorbic acid addition. Distinct lowercase letters on the bars indicate significant differences determined through statistical analysis among the samples (*p* ≤ 0.05).

In general, the temperature and duration of sterilization conditions in this study did not influence the physical properties of the texture-modified foods. However, the addition of ascorbic acid significantly impacted their hardness. The hardness, adhesiveness, springiness, and cohesiveness values of the samples are depicted in Figure 2. In Figure 2a, the sample treated at 121 °C for 37 min (121/37) exhibited the highest hardness value, followed by the samples treated at 125 °C for 11 min (125/11), 110 °C for 109 min (110/109), 125 °C for 11 min with ascorbic acid addition (125/11 [Vit C]), 110 °C for 109 min with ascorbic acid addition (110/109 [Vit C]), and 121 °C for 37 min with ascorbic acid addition (121/37 [Vit C]), respectively. The hardness values for all sterile samples without ascorbic acid did not significantly differ (*p* > 0.05) from the unsterilized samples. Conversely, the hardness values of all sterilized samples with added ascorbic acid were notably lower than those of the unsterilized samples (*p* ≤ 0.05), likely due to the shortened κ-carrageenan structure in the acidified foods during sterilization. Earlier reports have demonstrated that the heating process under acidic conditions can break the bonds between κ-carrageenan monomers [29,30].

All sterilized samples, as well as the unsterilized sample, showed no statistically significant difference in adhesiveness (*p* ≤ 0.05) (Figure 2b).

For samples without the addition of ascorbic acid, it was observed that high-temperature sterilization did not influence the hardness, adhesiveness, springiness, or cohesiveness of the gel (Figure 2). However, when the sample was supplemented with ascorbic acid, the statistical analysis indicated a notable decrease in gel strength. The inclusion of ascorbic acid in κ-carrageenan-containing gel before high-temperature sterilization can lead to a reduction in gel strength due to its reducing properties. Ascorbic acid has the ability to reduce the disulfide bonds that crosslink κ-carrageenan molecules, resulting in a weaker gel structure. The high-temperature sterilization process can also contribute to the weakening of the gel structure by breaking down κ-carrageenan molecules through hydrolysis and oxidation reactions. Therefore, the combination of ascorbic acid and high-temperature sterilization can lead to a significant reduction in the strength of κ-carrageenan-containing gels.

#### 3.2.2. Viscosity of Samples after the Sterilization Process

**Figure 3:**
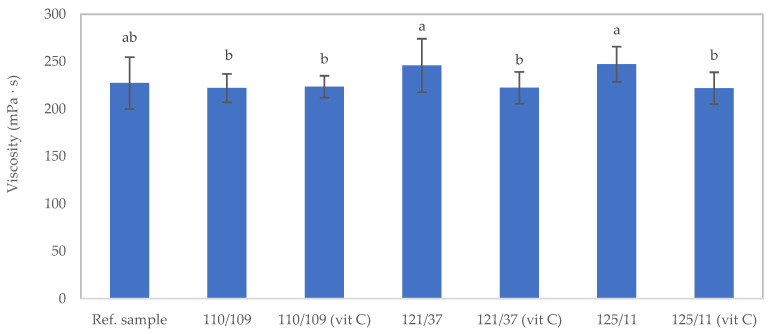
Viscosity of the texture-modified foods following sterilization under different conditions at a shear rate of 50 s^−1^: 110/109: 110 °C for 109 min without ascorbic acid; 121/37: 121 °C for 37 min without ascorbic acid; 125/11: 125 °C for 11 min without ascorbic acid; 110/109 (Vit C): 110 °C for 109 min with 0.1% (*w*/*v*) ascorbic acid addition; 121/37 (Vit C): 121 °C for 37 min with 0.1% (*w*/*v*) ascorbic acid addition; 125/11 (Vit C): 121 °C for 11 min with 0.1% (*w*/*v*) ascorbic acid addition. Distinct lowercase letters on the bars signify significant differences determined through statistical analysis among the samples (*p* ≤ 0.05).

The temperature and duration of sterilization conditions in this study, as well as the addition of ascorbic acid in the texture-modified foods, had minimal impact on the viscosity of the foods, as depicted in Figure 3. The viscosity of the samples after sterilization was generally not significantly different (*p* > 0.05) compared to the corresponding unsterilized sample, with the exception of the two conditions at 121 °C for 37 min and 125 °C for 11 min.

#### 3.2.3. Appearance

**Figure 4:**
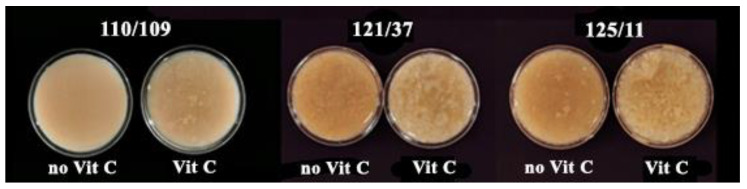

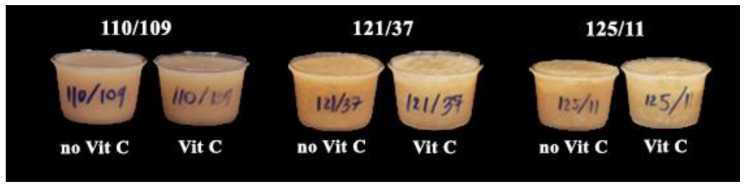
Appearance of the samples following sterilization under different conditions: 110/109: 110 °C for 109 min without ascorbic acid; 121/37: 121 °C for 37 min without ascorbic acid; 125/11: 121 °C for 11 min without ascorbic acid; 110/109(Vit C): 110 °C for 109 min with 0.1% (*w*/*v*) ascorbic acid addition; 121/37 (Vit C): 121 °C for 37 min with 0.1% (*w*/*v*) ascorbic acid addition; 125/11 (Vit C): 121 °C for 11 min with 0.1% (*w*/*v*) ascorbic acid addition.

**Figure 5:**
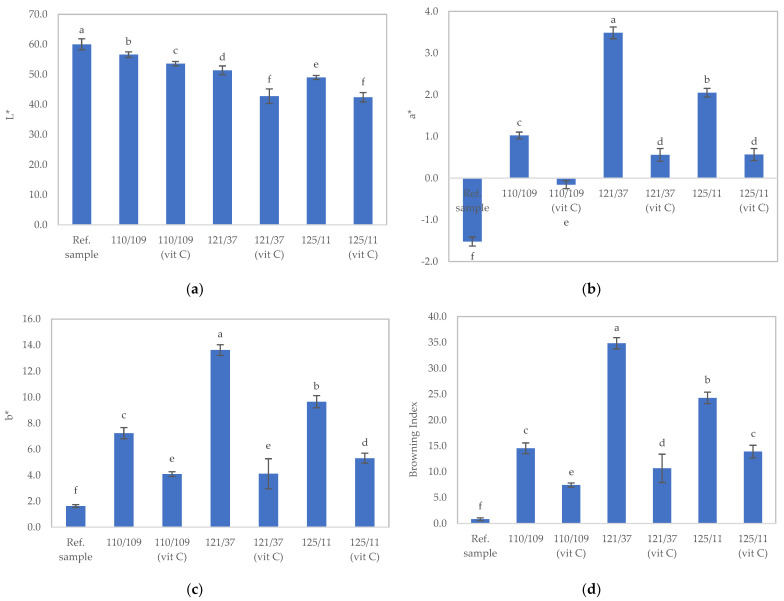
Color values of texture-modified foods after sterilization under different conditions: (**a**) L value, (**b**) a* value), (**c**) b* value, and (**d**) browning index. Sterilization conditions: 110/109: 110 °C for 109 min without ascorbic acid; 121/37: 121 °C for 37 min without ascorbic acid; 125/11: 121 °C for 11 min without ascorbic acid; 110/109 (Vit C): 110 °C for 109 min with 0.1% (*w*/*v*) ascorbic acid addition; 121/37 (Vit C): 121 °C for 37 min with 0.1% (*w*/*v*) ascorbic acid addition; 125/11 (Vit C): 121 °C for 11 min with 0.1% (*w*/*v*) ascorbic acid addition. Distinct lowercase letters on bars indicate significant differences determined through statistical analysis among the samples (*p* ≤ 0.05).

The physical appearance and color of the samples after sterilization were assessed, and the results are presented in Figure 4 and Figure 5a–d. Post sterilization, all sterilized samples exhibited significantly higher values in a*, b*, and the browning index compared to the unsterilized sample (*p* ≤ 0.05), attributable to the Maillard reaction between reducing sugars and amino acids during the heat processing of the foods. Moreover, sterilized samples with added ascorbic acid (110/109 [Vit C], 121/37 [Vit C], and 125/11 [Vit C]) displayed significantly lower values in a*, b*, and the browning index than samples lacking ascorbic acid addition (110/109, 121/37, and 125/11), as the presence of ascorbic acid could mitigate the Maillard reaction. Ascorbic acid is widely employed as an anti-browning agent in food production processes [31]. However, the appearance of all sterilized samples, except for those treated at 110 °C for 109 min (without ascorbic acid), would be deemed unsatisfactory due to sedimentation resulting from the denaturation of milk proteins in the samples (Figure 4).

Nonetheless, a high-temperature short-time (HTST) treatment, characterized by brief exposure to high temperatures, affects the browning reaction to a lesser extent than prolonged exposure to lower temperatures. The Maillard reaction, accountable for food browning, hinges on both time and temperature. The reaction advances more rapidly at higher temperatures, completing in a shorter duration. Conversely, lower temperatures lead to slower progression, necessitating an extended duration for completion. Consequently, HTST treatment results in a diminished timeframe for the Maillard reaction, leading to reduced browning in the food in comparison to extended exposure to lower temperatures.

#### 3.2.4. Sterilization Efficiency

The results indicate that all sterilized samples displayed bacterial counts below 30 CFU/g, adhering to the safety standards for consumer consumption as mandated by the Thai Public Health Ministry. Particularly, hermetically sealed containers are mandated to maintain bacterial counts lower than 1000 CFU/g. The elevated temperatures employed in this study (exceeding 100 °C for a specified duration across all sterilization conditions) were found to be highly efficient in eliminating nearly all bacteria present in the food samples.

### 3.3. Stability Study

**Figure 6:**
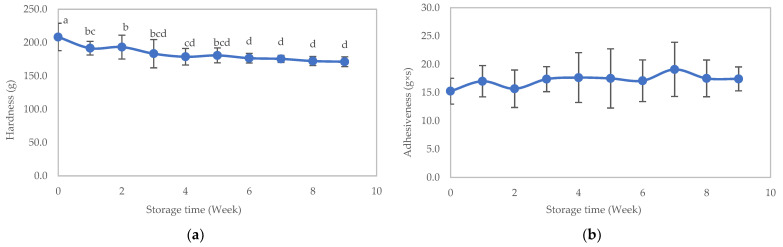

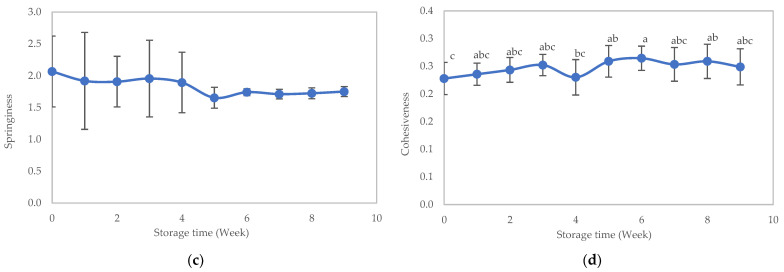
Texture profile analysis of the texture-modified foods during storage: (**a**) hardness, (**b**) adhesiveness, (**c**) springiness, and (**d**) cohesiveness. Distinct lowercase letters on the bars indicate significant differences determined through statistical analysis among the samples (*p* ≤ 0.05).

**Figure 7:**
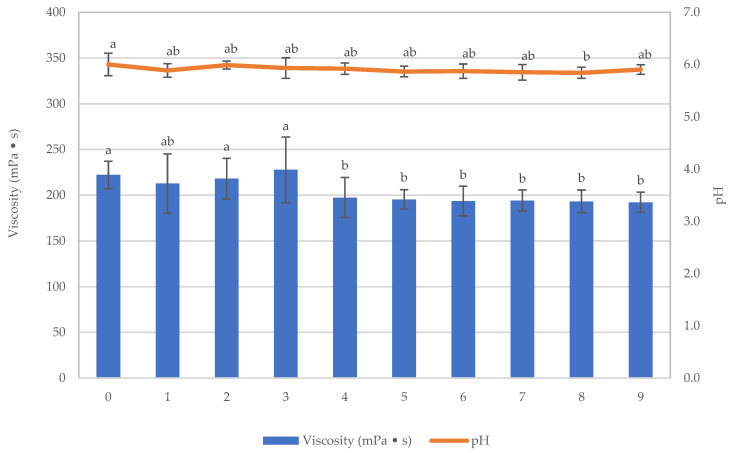
Viscosity and pH of the texture-modified foods during storage. Distinct lowercase letters on the bars signify significant differences determined through statistical analysis among the samples (*p* ≤ 0.05).

The results of the Texture Profile Analysis for the food are presented in Figure 6a–d. After storing the sample for 9 weeks and conducting assessments of physical quality (including hardness, adhesiveness, springiness, cohesiveness, viscosity, lightness, yellowness, redness, and browning index), chemical quality (including pH), and microbial quantity, it was observed that the sample’s hardness significantly decreased after being stored for 4 weeks, while adhesiveness displayed an increasing trend, though this effect was not statistically significant. Springiness and cohesiveness remained relatively consistent throughout the storage period. The viscosity of the texture-modified foods remained stable during the initial 3 weeks, followed by a gradual decline in week 4. Subsequently, it remained steady from weeks 4 to 9 (*p* > 0.05), as depicted in Figure 7.

The significant statistical reduction in sample viscosity observed after 4 weeks of storage could be attributed to the fact that in the initial stages of sample preparation, κ-carrageenan existed in a random coil conformation. Additionally, due to the negative charge of κ-carrageenan from sulfate groups within its molecular structure, it can form bonds with certain positively charged regions on the surface of the casein micelle, primarily with casein. This leads to aggregation. Subsequently, as the κ-carrageenan–casein mixture cools down, κ-carrageenan undergoes a change in its structure into a helical conformation [32], resulting in the formation of a gel-like structure in the solution, which entraps casein within the κ-carrageenan network. However, as time passes during extended storage at a lower temperature (4 ± 1 °C) for an extended period, the helical conformation of κ-carrageenan tends to self-aggregate more tightly, leading to the separation of casein [33]. The structural deterioration of the network results in a reduction in the overall system’s viscosity.

**Figure 8:**
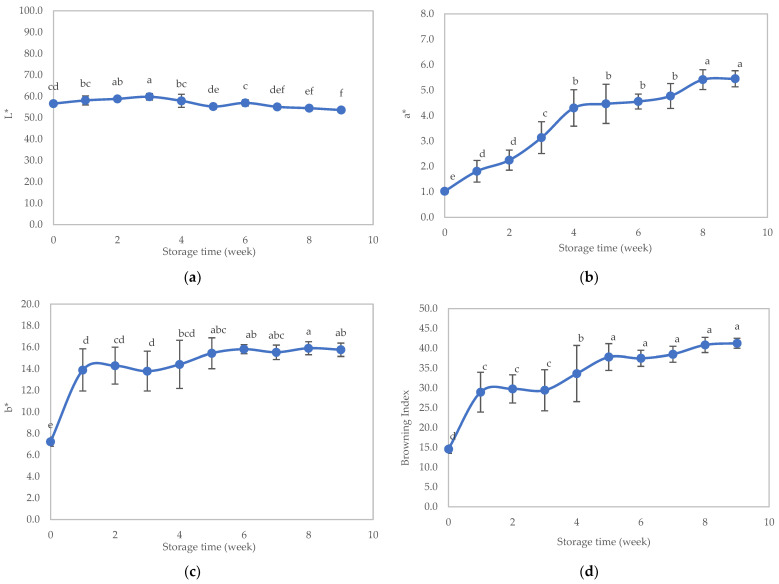
Color of the texture-modified foods during storage: (**a**) L value, (**b**) a* value, (**c**) b* value, and (**d**) browning index. Distinct lowercase letters on the bars signify significant differences determined through statistical analysis among the samples (*p* ≤ 0.05).

Regarding appearance, the L* value of the sample remained steady during the initial 4 weeks but exhibited a statistically significant decrease thereafter, while the a* value, b* value, and browning index displayed an ascending trend throughout the storage duration (Figure 8, panels a–d).

In summary, the product exhibited a slightly softer texture and a slightly darker color due to the Maillard reaction, yet the pH value remained unchanged (Figure 7) and no bacterial presence was detected during the storage period. One of the principal transformations that can transpire in κ-carrageenan-containing food during prolonged refrigeration is a decline in texture and consistency. κ-Carrageenan is a hydrocolloid, serving as a thickening and stabilizing agent in food items. Over time, κ-carrageenan may degrade, resulting in diminished thickening attributes and consequent alterations in the food’s texture and consistency. This, in turn, can potentially render the food less appealing to consumers.

## 4. Conclusions

The sample obtained from the experiment possessed a moderately soft and chewable texture, without syneresis. Increasing the concentration of the hydrocolloid notably enhanced the gel strength (hardness) of the sample. Xanthan gum, at all studied concentrations, as well as low concentrations of glucomannan and gelatin, were unable to form a gel in the texture-modified foods. Among the hydrocolloids at the same concentrations, the texture-modified foods treated with κ-carrageenan exhibited the highest hardness value. However, the texture-modified foods treated with 1% κ-carrageenan closely match the properties of hospital jelly samples in terms of hardness, adhesiveness, and viscosity for training patients with level 4 and 5 (IDDSI standards) swallowing problems. The food was devised with appropriate sterilization conditions, utilizing a retort at 110 °C for 109 min, without the inclusion of ascorbic acid. When the sample was supplemented with ascorbic acid, the statistical analysis indicated a notable decrease in gel strength after high-temperature sterilization. This approach yielded physical properties akin to those of unsterilized samples, along with a limited bacterial count, rendering it a safe dietary option. The texture-modified food treated under these conditions demonstrated favorable hardness and exhibited acceptable browning changes during storage at room temperature (30 + 2 °C) for up to 9 weeks. All sterilization procedures effectively eliminated bacteria.

## Figures and Tables

**Table 1:** Types and concentrations of hydrocolloids.

Types of Hydrocolloids	Level of Addition		
	1%	3%	5%
Agar	A1	A3	A5
κ-Carrageenan	C1	C3	C5
Gelatin	G1	G3	G5
Konjac Glucomannan	K1	K3	K5
Xanthan gum	X1	X3	X5

**Table 2:** Texture profile analysis of texture-modified foods treated with various hydrocolloids.

Sample	Hardness (g)	Adhesiveness (g·s)	Springiness	Cohesiveness
Richese^®^ (Ref. sample)	238.29 ± 21.72 ^c^	3.28 ± 1.19 ^e^	0.9 ± 0.41 ^g^	0.29 ± 0.04 ^d^
A1	45.57 ± 2.37 ^e^	44.86 ± 21.89 ^d^	6.33 ± 0.36 ^b^	0.35 ± 0.03 ^c^
A3	77.83 ± 2.88 ^e^	145.09 ± 10.58 ^b^	1.18 ± 0.10 ^fg^	0.28 ± 0.02 ^d^
A5	211.47 ± 3.08 ^cd^	232.88 ± 35.83 ^a^	8.15 ± 0.81 ^a^	0.38 ± 0.03 ^bc^
C1	221.72 ± 13.36 ^cd^	11.63 ± 1.58 ^e^	2.52 ± 0.53 ^d^	0.18 ± 0.02 ^f^
C3	329.15 ± 35.19 ^b^	46.29 ± 14.04 ^d^	3.02 ± 0.73 ^c^	0.23 ± 0.02 ^e^
C5	814.74 ± 169.45 ^a^	50.09 ± 13.43 ^d^	1.83 ± 0.17 ^e^	0.24 ± 0.02 ^e^
G1	NA	NA	NA	NA
G3	71.63 ± 11.93 ^e^	49.30 ± 5.95 ^d^	1.47 ± 0.44 ^ef^	0.35 ± 0.41 ^c^
G5	163.85 ± 15.69 ^d^	114.29 ± 29.07 ^c^	1.31 ± 0.32 ^fg^	0.40 ± 0.41 ^b^
K1	NA	NA	NA	NA
K3	NA	NA	NA	NA
K5	54.87 ± 0.72 ^e^	160.59 ± 13.67 ^b^	1.14 ± 0.16 ^fg^	0.71 ± 0.04 ^a^
X1	NA	NA	NA	NA
X3	NA	NA	NA	NA
X5	NA	NA	NA	NA

Please refer to the sample abbreviations in Table 1. All data are presented as the mean ± standard deviation (SD) from triplicate determinations (*n* = 3). Distinct superscripts within the same column indicate significant differences determined through statistical analysis among the samples (*p* ≤ 0.05). “NA” denotes that the sample did not solidify into a gel, making it unfeasible to conduct a texture profile analysis.

**Table 3:** Physical characteristics of gel formation and syneresis.

Physical Characteristics of Gel Formation and Syneresis	Sample
Soft gel with syneresis	A1, G1
Soft gel without syneresis (target texture characteristics)	K5, C1
Hard gel with syneresis	G3, G5
Hard gel without syneresis	A3, A5, C3, C5
Viscous solution (no gel formed)	K1, K3, X1, X3, X5

## Data Availability

The data are available by reasonable request to the corresponding author.
